# Diagnostic and Therapeutic Implications of microRNAs in Non-Small Cell Lung Cancer

**DOI:** 10.3390/ijms21228782

**Published:** 2020-11-20

**Authors:** Young-Ho Ahn, Yoon Ho Ko

**Affiliations:** 1 Department of Molecular Medicine, College of Medicine, Ewha Womans University, Seoul 07804, Korea; 2 Inflammation-Cancer Microenvironment Research Center, College of Medicine, Ewha Womans University, Seoul 07804, Korea; 3 Division of Oncology, Department of Internal Medicine, College of Medicine, The Catholic University of Korea, Seoul 06591, Korea; 4 Cancer Research Institute, College of Medicine, The Catholic University of Korea, Seoul 06591, Korea

**Keywords:** microRNA, non-small cell lung cancer, diagnosis, prognosis

## Abstract

microRNAs (miRNAs), endogenous suppressors of target mRNAs, are deeply involved in every step of non-small cell lung cancer (NSCLC) development, from tumor initiation to progression and metastasis. They play roles in cell proliferation, apoptosis, angiogenesis, epithelial-to-mesenchymal transition, migration, invasion, and metastatic colonization, as well as immunosuppression. Due to their versatility, numerous attempts have been made to use miRNAs for clinical applications. miRNAs can be used as cancer subtype classifiers, diagnostic markers, drug-response predictors, prognostic markers, and therapeutic targets in NSCLC. Many challenges remain ahead of their actual clinical application; however, when achieved, the use of miRNAs in the clinic is expected to enable great progress in the diagnosis and treatment of patients with NSCLC.

## 1. Introduction

microRNAs (miRNAs) are small, non-coding RNAs that are 20–25 nucleotides in length [1,2], and are endogenous suppressors of target genes [3]. They have complementary sequences to 3′-untranslated regions (3′-UTRs) of target mRNAs and bind to these regions through Watson–Crick base pairing. Perfectly matched binding between miRNAs and 3′-UTRs leads to mRNA cleavage and degradation, while imperfectly matched binding leads to translational repression. This binding involves 7–8 nucleotides of the miRNAs, which is called the seed sequence; therefore, one miRNA can target multiple mRNAs, and one mRNA can be targeted by multiple miRNAs. Through suppressing target genes, miRNAs regulate diverse physiological and pathological conditions, including cancer. miRNAs can either promote or repress cancer development and progression according to their target genes. Numerous miRNAs can act as oncogenes by negatively regulating tumor suppressors. For example, miR-21 expression is up-regulated in colon cancer, and promotes cell growth and invasion by repressing the tumor suppressor *PTEN* [4]. Conversely, let-7 inhibits cellular proliferation by negatively regulating the *KRAS* oncogene in lung cancer [5].

Lung cancer manifests as a malignant tumor caused by uncontrolled cell growth in the bronchiolar and alveolar epithelium of the lungs. Lung cancer is the most common cancer worldwide in both incidence (11.6% of the total cases) and mortality (18.4% of the total cancer deaths) [6]. Long-term smoking is well-known to be the main cause of lung cancer, while environmental effects (e.g., air pollution and particulate matters) and genetic variations (e.g., *KRAS* and *EGFR* mutations) can also cause lung cancer [7]. Lung cancer is classified into two main types depending on their histological phenotype: small cell lung cancer (SCLC) and non-small cell lung cancer (NSCLC) [7]. SCLC accounts for 15% of lung cancer, and is mainly associated with smoking. NSCLC accounts for 85% of lung cancer, and is further divided into adenocarcinoma (LUAD), squamous cell carcinoma (LUSC), and large cell carcinoma on the basis of cellular pathology and sites of origin. This review focuses on NSCLC, and describes the latest research results and trends regarding the clinical implications of using miRNAs for the treatment of NSCLC.

## 2. Biogenesis of miRNAs

Since the first miRNA, *lin-4*, was discovered in *Caenorhabditis elegans* in 1993 [8,9], thousands of miRNAs have been identified in both animals and plants due to the development of molecular genetics and next-generation sequencing technology. According to miRBASE (Release 22.1, October 2018; http://www.mirbase.org), 2656 mature miRNAs have been identified in the human genome to date [10]. miRNAs are encoded in the genome as single genes (i.e., monocistronic) or as clusters that are transcribed together with other miRNAs (i.e., polycistronic). miRNAs are generally transcribed by RNA polymerase II in the nucleus in a form called primary miRNAs (pri-miRNAs), which can be longer than thousands of nucleotides. As RNA polymerase II is involved, the transcription of pri-miRNAs is regulated by general transcription factors and signal transduction pathways. The pri-miRNAs are also capped at the 5′-end and polyadenylated at the 3′-end, just like mRNA transcripts.

Like normal single-stranded RNA molecules, pri-miRNA can form secondary structures in which part(s) of the pri-miRNA strand forms hairpin loops, stabilized by intramolecular hydrogen bonds. The mature miRNAs, comprised of 20–25 nucleotides, are contained in the stem of the hairpin structures. Once transcription is completed, pri-miRNAs are processed into premature miRNAs (pre-miRNAs) which are stem–loop structures of about 80 nucleotides in length. The processing of pri-miRNAs to pre-miRNAs is fulfilled by a microprocessor complex, which consists of Drosha, a double-stranded RNA-specific ribonuclease (RNase) III [1,2,11,12], and DGCR8, a molecular anchor recognizing the double-strand/single-strand junction of pri-miRNAs [13]. With the help of other additional factors, such as DDX5 and DDX17 RNA helicases [14], the Drosha:DGCR8 complex cuts out the stem–loop structure (pre-miRNA) from the pri-miRNA at the precise positions on the 5′ and 3′ sides.

This processing of pri-miRNAs to pre-miRNAs occurs in the nucleus. Processed pre-miRNAs are exported to the cytoplasm by exportin 5, a double-stranded RNA-binding protein, and RanGTP, a GTP-binding nuclear protein [1,15,16,17]. Exportin 5 binds to stem–loop pre-miRNAs, and RanGTP binding triggers nuclear export of the pre-miRNA:exportin 5 complex. Pre-miRNAs are transported through the nuclear pore and then released into the cytoplasm upon the hydrolysis of RanGTP to RanGDP. Intriguingly, exportin 5 has also been reported to promote pri-miRNA processing, which is independent of RanGTP [18].

Once released into the cytoplasm, the stem of the pre-miRNA is further processed to a small RNA duplex (20–25 nucleotides) by another RNase III-type endonuclease, Dicer. The N-terminal helicase domain of Dicer recognizes pre-miRNAs at their terminal loop, and the internal PAZ domain binds to the termini of pre-miRNAs. The recognition of pre-miRNAs by the helicase and PAZ domains may function as “a molecular ruler” which guarantees precise cleavage of pre-miRNAs [1]. Dicer cooperates with the RNA-binding protein TRBP, which facilitates pre-miRNA processing and determines the exact length of mature miRNAs [19].

Processed miRNA duplexes bind to Ago proteins, which are main components of the RNA-induced silencing complex (RISC). During this process, only one of the two miRNA strands is specifically selected (guide strand) to function as a mature miRNA, while the other is degraded (passenger strand). The exact mechanism of strand selection has not been fully elucidated; however, it has been shown that stability of duplex ends and types of 5′-terminal nucleotides can determine strand fate [20]. In some cases, both the 5′- (5p) and 3′-sides (3p) of a pre-miRNA can be functional [21]. For example, both miR-142-5p and miR-142-3p are downregulated in liver cancer possibly through promoter hypermethylation [22] and synergistically suppress cancer cell migration by regulating actin cytoskeleton, adherens junctions, and focal adhesion [23]. Furthermore, “arm switching” between two strands of pre-miRNA duplexes can occur during cancer development, which can be used as a cancer biomarker [20].

## 3. Functional Mechanisms of miRNAs

In most cases, miRNAs recognize mRNA targets via binding to specific sequences at their 3′-UTRs; however, some miRNAs can bind to 5′-UTRs or coding sequences of mRNA targets [24]. For example, miR-1254 interacts with the 5′-UTR of *CCAR1* and enhances its stability [25], while miR-20a represses *DAPK3* by binding to its protein coding sequences (exon 2) [26]. Even promoter regions can be targeted by miRNAs; let-7i binds to the core promoter region of *IL2* and up-regulates *IL2* promoter activity [27]. Recently, numerous studies have shown that long non-coding RNAs are also binding partners of miRNAs [28].

Mature miRNAs loaded onto the RISC mediate gene silencing via two mechanisms: mRNA decay and translational repression [29,30]. Ago proteins have an RNase H-like domain; thus, RISC can cleave mRNA targets when they are perfectly complementary to miRNAs, which is common in plants [31]. In contrast, animal miRNAs bind to their target mRNAs at seed sequences of 7–8 nucleotides in length thorough partial complementarity, which instead induces translational repression and mRNA decay. miRNA-loaded Ago proteins bind to GW182, a scaffolding protein [32] that recruits poly(A)-binding protein (PABP) and deadenylase complexes (CCR4-NOT and PAN2-PAN3). Deadenylation then causes degradation of target mRNAs. In addition, RISC recruits decapping factors, such as DCP1, DCP2, and DDX6, which promotes decapping and degradation of target mRNAs [29,30].

**Figure 1:**
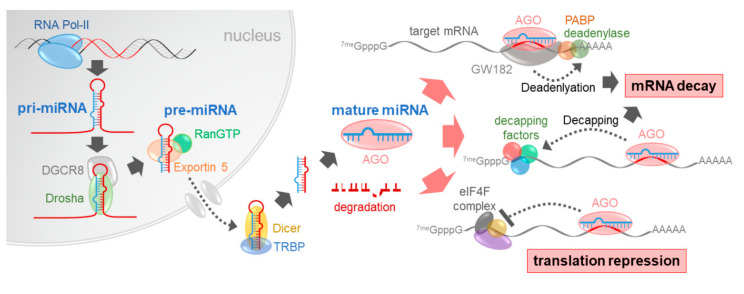
Biogenesis and functional mechanisms of miRNAs.

GW182 binding to RISC represses initiation of translation by breaking the interaction between PABP and eIF4G, which stimulates ribosome recruitment [29]. GW182 also recruits translation repressors working at the inhibition step, such as DDX6 [33] and eIF4E-binding protein 4E-T [34]. In addition, miRNAs have been shown to dissociate the eIF4A complex, thus inhibiting ribosome binding and scanning [35]. Translational repression and mRNA decay are both crucial mechanisms of miRNA-mediated gene silencing; however, these two mechanisms do not always work synergistically [36], and the preference between the two mechanisms may depend on cellular context or miRNA:mRNA binding characteristics [29,37]. The biogenesis and functional mechanisms of select miRNAs are summarized in Figure 1.

## 4. Regulation of miRNA Expression

miRNA expression can be regulated through transcriptional and post-transcriptional mechanisms [38]. Like mRNAs transcribed by RNA polymerase II complex, miRNAs are also under the transcription control of various signaling pathway components and transcription factors [39]. Promoter methylation is also one of major mechanisms for regulating miRNA expression. miR-34a is downregulated in colon cancers with liver metastases, which is due to hypermethylation on the promoter region [40]. Upregulation of oncogenic miR-21 in various types of cancer is associated with hypomethylation on the promoter region [41]. In addition, histone modification on the promoter of miR-200b/200a/429 cluster causes silencing of miR-200 family members and promotes stemness of breast cancer cells [42].

After transcription and processing/maturation, miRNA levels are further regulated by diverse endogenous factors. Ago2 protein increases miRNA abundance by promoting miRNA processing and enhancing miRNA stability, which is independent of its RNase function [43]. RNA binding protein FXR1 stabilizes miR-301a-3p and facilitates p21 targeting in oral cancer [44]. QKI-5 directly interacts with miR-196b-5p and reduces its stability [45]. Numerous competing endogenous RNAs (ceRNAs) are also involved in the regulation of miRNA levels. *ITGA1* mRNA functions as a sponge against miR-181b and relieves *ADCY9* targeting [46]. *FN1* mRNA acts as a ceRNA for miR-200c and modulates epithelial-to-mesenchymal transition (EMT) in breast cancer cells [47]. Moreover, one of the main features of long non-coding RNAs [48] and circular RNAs [49] is their functioning as ceRNAs, thus preventing the interaction between miRNAs and their target genes.

## 5. Roles of miRNAs in Progression and Metastasis of NSCLC

From the moment of tumor initiation to distant metastasis, epithelial cancer cells, such as NSCLC cells, must undergo a series of steps that are necessary for the acquisition of their pathological properties [50]. First, certain epithelial cells gain a selective growth advantage via genetic or epigenetic events. Through the EMT, highly proliferative cancer cells lose cell-to-cell and cell-to-matrix adhesion, and separate from the primary tumor. These cells acquire additional invasive abilities, and invade into basement membranes and stromal extracellular matrix (ECM). The invasive cancer cells further invade into surrounding blood or lymphatic vessels (intravasation). Then, they are transported via the circulatory system to distant sites and exit from blood vessels (extravasation). Once at the new site, the cancer cells reinvade, adapt to the new environment, and, finally, proliferate to form secondary metastatic tumors. miRNAs are closely involved in every step of NSCLC progression and metastasis, and precisely control the expression and activity of key factors during these processes. Select miRNAs that are known to regulate NSCLC development are shown in Table 1.

### 5.1. Primary Tumor Growth

Abnormal and uncontrolled cell growth beyond defined boundaries is the initial and essential step of tumor progression. Both enhanced proliferation and suppressed cell death (apoptosis) increase cancer cell growth and are controlled by numerous miRNAs targeting oncogenes or tumor suppressors. let-7 family members inhibit proliferation of NSCLC cells by directly targeting K-Ras [51] and cyclin D1 [52]. miR-34 family members (miR-34a, 34b, and 34c) also suppress NSCLC cell proliferation by targeting cyclin E1 [53], CDK4 [54], and c-Myc [55]. In contrast, miR-224 promotes NSCLC growth by targeting TNFα-induced protein 1, which is involved in DNA synthesis and apoptosis [56]. miR-212 also exerts tumor-promoting effects in NSCLC cells via suppression of the hedgehog signaling pathway receptor PTCH1 [57]. miR-21, a well-known oncogenic miRNA, promotes NSCLC progression by targeting the tumor suppressor PTEN [58]. Apoptosis is also modulated by miRNAs. miR-34a and miR-7 target BCL-2 [59,60], and miR-195 targets survivin [61], both of which are anti-apoptotic proteins. In contrast, miR-484 targets Apaf-1 [62], and miR-182 and miR-494 target caspase-2, both of which are key promoters of apoptosis [63,64].

### 5.2. Angiogenesis and Hypoxia

The formation of a network of new blood vessels that can supply enough oxygen and nutrients to the tumor tissues is essential for optimal tumor outgrowth. Cancer cells facilitate growth of vascular endothelial cells and promote angiogenesis by secreting vascular endothelial growth factors (VEGFs) [50]. miR-128 and miR-195 directly target VEGFs thereby suppressing tumorigenesis and angiogenesis [65,66]. miR-200b targets the VEGF receptors Flt-1 and KDR, and suppresses cancer cell invasion and metastasis [67,68]. On the opposite side, miR-130b promotes tumorigenesis by targeting TIMP-2, an inhibitor of metalloproteinase-2 and angiogenesis [69]. An inadequate supply of oxygen around the tumor microenvironment causes hypoxia. HIF1α, which promotes angiogenesis, is induced under hypoxic conditions. Several studies have reported that HIF1α is also targeted by several miRNAs, such as miR-130a, miR-199a, and miR-200c [70,71,72].

### 5.3. EMT, Migration, and Invasion

Epithelial cancer cells lose apical–basal polarity during EMT, and turn into mesenchymal-like cells with enhanced migratory and invasive capacities [73]. EMT is regulated by various EMT-inducing transcription factors (e.g., ZEB1/2, Snail, Slug, Twist), cell adhesion molecules (e.g., E-cadherin, N-cadherin), and tight junction proteins (e.g., Crumbs, Claudins), as well as numerous miRNAs. miR-200 family members that target ZEB1/2 are well-known suppressors of EMT [74]. Moreover, Snail and Twist are direct targets of miR-34a [75] and miR-98 [76], respectively. miR-544a promotes invasion by targeting E-cadherin [77], and miR-124 suppresses EMT by targeting N-cadherin [78]. Matrix metalloproteinases (MMPs) also play key roles during cancer cell invasion and metastasis [79]. Among them, MMP2 is a target of miR-29b [80], and MMP14 is a target of miR-584 [81]. As mentioned above, TIMP-2, which regulates MMPs’ activity, is a target of miR-130b [69]. In addition, regulators of Rho GTPases, which are involved in actin cytoskeleton remodeling, filopodia and lamellipodia formation, adhesion, migration, and invasion of cancer cells, are also targeted by various miRNAs [82].

### 5.4. Survival and Immune Escape

Lack of attachment to the ECM triggers cell death (anoikis) in epithelial cells. Once they invade the vascular or lymphatic system, cancer cells need to acquire anoikis resistance [83]. miRNAs promote or inhibit anoikis by targeting key molecules involved in anoikis signaling pathways [84]. miR-34a and miR-451 enhance the susceptibility of lung cancer cells to anoikis [85,86]. miR-148a inhibits anchorage-independent growth by targeting MMP15 and ROCK1 in NSCLC [87]. In contrast, exosomal miR-222 promotes cell survival under anchorage-independent conditions by directly targeting SOCS3 [88]. Cancer cells must survive the attacks of the immune system to progress, and miRNAs are involved in both immune attacks on tumors and immune escape [89,90]. miR-451 suppresses cell proliferation and metastasis in lung cancer cells by directly targeting PSMB8, which is one subunit of an immunoproteasome and modulates inflammatory responses [91]. miR-200 family members target PD-L1 and control immunosuppression in NSCLC cells [92]. miR-138, miR-140, and miR-142 have also been reported to target PD-L1 [93,94,95]. On the contrary, miR-197 enhances PD-L1 expression through the regulation of the cyclin-dependent kinase CKS1B and STAT3 pathway [96].

### 5.5. MET and Metastatic Colonization

A subset of cancer cells that survive within the systemic circulation will ultimately colonize at a distant metastatic site. In contrast to the initial stages of cancer development, these cancer cells lose their mobility and invasiveness and regain cell-to-cell and cell-to-matrix adhesiveness, which is a reverse process of EMT known as the mesenchymal-to-epithelial transition (MET) [97]. Although metastasis occurs independently of MET in some cases [98], various miRNAs associated with EMT also regulate MET. Ectopic expression of miR-200 family members induces MET in highly metastatic lung cancer cells [99]. Additionally, miR-147 induces MET and reverses drug resistance [100]. miR-29b promotes MET and prevents lung fibrosis [101]. Selection of the final metastatic destination and colonization at various organ sites, such as the bones, brain, and lymph nodes, is also affected by a variety of miRNAs [102].

**Table 1:** miRNAs regulating progression and metastasis of non-small cell lung cancer.

miRNA	Type ^1^	Effect	Target Gene	References
**Tumor Growth and Apoptosis**				
let-7 family	tsmiR	Inhibits cell proliferation	*KRAS*, *CCND1*	[51,52]
miR-34 family	tsmiR	Inhibits cell proliferation, promotes apoptosis	*CCNE1*, *CDK4*, *MYC*, *BCL2*	[53,54,55] [59]
miR-7	tsmiR	Promotes apoptosis	*BCL2*	[60]
miR-195	tsmiR	Promotes apoptosis	*BIRC5*	[61]
miR-224	oncomiR	Promotes cell growth	*TNFAIP1*	[56]
miR-212	oncomiR	Promotes cell growth	*PTCH1*	[57]
miR-21	oncomiR	Promotes tumor progression	*PTEN*	[58]
miR-484	oncomiR	Suppresses apoptosis	*APAF1*	[62]
miR-182	oncomiR	Suppresses apoptosis	*CASP2*	[63]
miR-494	oncomiR	Suppresses apoptosis	*CASP2*	[64]
**Angiogenesis**				
miR-128	tsmiR	Suppresses angiogenesis	*VEGFA*	[65]
miR-195	tsmiR	Suppresses angiogenesis	*VEGFA*	[66]
miR-200b	tsmiR	Suppresses angiogenesis, invasion, and metastasis	*FLT1*, *KDR*	[67,68]
miR-130a	tsmiR	Suppresses angiogenesis	*HIF1A*	[70]
miR-199a	tsmiR	Suppresses angiogenesis	*HIF1A*	[71]
miR-200c	tsmiR	Suppresses angiogenesis	*HIF1A*	[72]
miR-130b	oncomiR	Promotes angiogenesis	*TIMP2*	[69]
**Epithelial-to-Mesenchymal Transition (EMT), Migration, and Invasion**				
miR-200 family	tsmiR	Inhibits EMT	*ZEB1*, *ZEB2*	[74]
miR-34a	tsmiR	Inhibits EMT	*SNAI1*	[75]
miR-98	tsmiR	Inhibits EMT	*TWIST1*	[76]
miR-544a	oncomiR	Promotes EMT	*CDH1*	[77]
miR-124	tsmiR	Inhibits EMT	*CDH2*	[78]
miR-29b	tsmiR	Inhibits invasion	*MMP2*	[80]
miR-584	tsmiR	Inhibits invasion	*MMP14*	[81]
miR-130b	oncomiR	Promotes invasion	*TIMP2*	[69]
**Anchorage-Independent Survival and Immune Escape**				
miR-148a	tsmiR	Inhibits cell survival	*MMP15*, *ROCK1*	[87]
miR-222	oncomiR	Promotes cell survival	*SOCS3*	[88]
miR-451	tsmiR	Inhibits immune escape	*PSMB8*	[91]
miR-200 family	tsmiR	Inhibits immune escape	*CD274*	[92]
miR-138	tsmiR	Inhibits immune escape	*CD274*	[95]
miR-140	tsmiR	Inhibits immune escape	*CD274*	[93]
miR-142	tsmiR	Inhibits immune escape	*CD274*	[94]
miR-197	oncomiR	Promotes immune escape	*CSK1B*	[96]
**Mesenchymal-to-Epithelial Transition (MET) and Colonization**				
miR-200 family	tsmiR	Promotes MET	*ZEB1*, *ZEB2*	[99]
miR-29b	tsmiR	Promotes MET	*TGFB1*	[101]

^1^ tsmiR: tumor suppressive miRNA, oncomiR: oncogenic miRNA.

## 6. Clinical Implications of miRNAs in NSCLC

Considering that multiple miRNAs have functions similar to their target genes and that a single miRNA can regulate several mRNAs, a panel of miRNAs is considered a better biomarker than individual miRNAs for clinical applications [74]. Since cancer-associated miRNA biomarkers can be easily detected in tissue, blood, or other bodily fluids, circulating miRNAs grant several potential advantages for clinical application, including high stability in serum, ease of non-invasive detection in circulation, and a convenient screening method [103]. Circulating miRNAs also afford a chance to overcome the problem of tumor heterogeneity by allowing for the collection of all pathological signals from many disparate portions of primary tumors and metastatic sites.

### 6.1. NSCLC Subtype Classifiers

LUAD and LUSC are the major histological subtypes of NSCLC. LUSC is most commonly associated with tobacco use, while LUAD is commonly associated with non-smokers and women [104], suggesting the existence of several underlying major differences not only in biological patterns, but also in molecular characteristics, between histological subtypes. For example, activating mutations in epidermal growth factor receptor (EGFR) and mutations in anaplastic lymphoma kinase (ALK) fusion proteins usually occur in LUAD, but not in LUSC, rendering therapy targeted at these genes ineffective for LUSC [105]. MiRNAs can be used to distinguish subtypes among NSCLC. In a previous report, four miRNAs (miR-205, miR-93, miR-221, and miR-30e) were shown to be highly expressed in LUSC, and five miRNAs (miR-29b, miR-29c, let-7e, miR-100, and miR-125a-5p) were highly expressed in LUAD [106]. Through an analysis of three miRNome profiling datasets, Hu et al. identified that miR-375, miR-203, and miR-205 were differentially expressed miRNAs that could be used to distinguish LUSC from other NSCLC subtypes [107]. A recent study using machine learning approaches identified miR-944 and miR-205 as useful for classifying tumors into the LUAD and LUSC subtypes [108].

### 6.2. Diagnostic Markers

To date, serum tumor markers have not been employed for early lung cancer screening due to limitations in their effectiveness, sensitivity, and specificity. However, circulating miRNAs have demonstrated potential advantages for use in in clinical screening methods [103]. Currently, several articles have shown that many kinds of circulating miRNAs can be used to detect lung cancer [109,110,111,112]. Recently, in an analysis of serum samples from 1566 lung cancer and 2178 non-cancer participants, the diagnostic accuracy, sensitivity, and specificity of the combined expression levels of two miRNAs (miR-1268b and miR-6075) were all 99%, regardless of the histological type and pathological Tumor, Node, Metastasis (TNM) stage of the NSCLC [110]. In another study of 2856 participants, a 14-miRNA signature distinguished patients with lung cancer from patients with non-tumor lung diseases with an accuracy of 92.5%, sensitivity of 96.4%, and specificity of 88.6%. In addition, the expression level of miR-17-3p distinguished patients with lung cancer from those with non-tumor lung diseases with the highest significance and an Area Under the Receiver Operating Characteristics (AUROC) value of 0.899 [112].

### 6.3. Drug-Response Predictors

Furthermore, miRNAs could be useful for predicting tumor responsiveness to chemotherapy or different therapeutic approaches. In a study with drug-resistant NSCLC cell lines, several markers were identified as being predictive of the degree of responsiveness to therapy, with miR-192, miR-194, miR-205, miR-30a, and miR-30c demonstrated to be predictive factors for a positive response to chemotherapy [113]. In an analysis of 148 LUAD patients who were negative for EGFR mutations or ALK translocations and who received maintenance treatment with pemetrexed, progression-free survival duration for patients expressing different levels of circulating miR-25, miR-145, and miR-210 were significantly different in the pemetrexed-treated group, suggesting these three miRNAs are predictors for the efficacy of maintenance treatment [114]. Recently, with the advent of immune checkpoint blockade therapy, immunotherapy has shown promising results in various types of cancer including lung cancer [115]. In a comparative analysis between responders and non-responders to PD-1/PD-L1 inhibitors, miR-320 family members, such as miR-320d, miR-320c, and miR-320b, were identified as potential biomarkers for predicting the efficacy of immunotherapy in advanced NSCLC. In addition, the level of exosomal miR-125b-5p was dramatically downregulated in the partial response-post samples, indicating that miR-125b-5p levels might be useful for monitoring the efficacy of anti-PD-1/PD-L1 treatment [116]. When the T-cell suppressor miR-125b-5p is downregulated during immunotherapy, patients may achieve increased T-cell function and respond well to immunotherapy. Of note, plasma-derived exosomes detected in patients are mainly released from tumor cells, which can more accurately and dynamically reflect the state and function of tumor cells.

### 6.4. Prognostic Markers

A prognostic biomarker should ideally provide information on the overall disease outcome in patients, such as disease recurrence or disease progression, independent of the treatment regimen. The discovery of prognostic factors could contribute to classifying patients by prognosis and identifying high-risk cases requiring aggressive approaches [117]. Similar to oncogenes and tumor suppressor genes, oncogenic miRNAs and tumor suppressive miRNAs are playing major roles in accurately identifying lung cancer prognoses. Numerous studies have shown that individual miRNAs play prognostic roles in NSCLC patients [52,54,58,61,74,76,77,78,80,91,96,99]. In a recent analysis using the TCGA database, two different prognostic miRNA signatures (a four-miRNA signature for LUAD: miR-375, miR-148a, miR-29b-1, and miR-584; and a four-miRNA signature for LUSC: miR-4746, miR-326, miR-93, and miR-671) were found to be independent prognostic factors in LUAD and LUSC patients [118]. Machine learning algorithms are indeed useful methods for analyzing large volumes of data, such as genetic information produced by next-generation sequencing technologies. We previously applied a neural network-based algorithm called Cascaded Wx framework to extract miRNA markers most highly associated with LUAD patient survival [119]. Through subsequent profiling of miRNA expression levels in LUAD patient samples, miR-374a and miR-374b, both EMT-related miRNAs, were identified as potential prognostic markers associated with poor survival in LUAD patients [120].

### 6.5. Therapeutic Targets

MiRNAs have various functions within cancer cells, with some having a high specificity for cancer-associated pathways, and they are one of the most promising therapeutic targets with well-characterized expressions. Biologically, the attractiveness of using miRNAs for cancer treatment comes from their ability to target multiple genes involved in multiple cancer-related pathways. Adding tumor suppressive miRNAs or reducing oncogenic miRNAs in cancer cells could be effective as a therapeutic strategy. In in vivo lung cancer models for therapeutic applications, miR-15/16 [121], miR-29b [122], miR-7 [123], miR-34a [124], let-7 [125], miR-200c [126], and miR-145 [127] were tested using different delivery systems. Two phase I clinical trials have been conducted for advanced solid cancers, including NSCLC (Table 2) [128,129,130]. The first-in-human, phase I study of a microRNA-based cancer therapy using MRX34, a liposomal mimic of miR-34a, was conducted but closed early due to unexpectedly severe immune-mediated toxicities with a modest overall response rate of 4% [129]. In contrast, in a phase I MesomiR-1 trial, TargomiRs, comprised of a miR-16-based microRNA mimic packaged in EDV^TM^ nanocells that are targeted with an anti-EGFR-specific antibody, was tested in patients with advanced NSCLC or malignant pleural mesothelioma. Overall, TargomiR treatment has been well tolerated and shown to be safe in patients. Interim data indicated that disease control was achieved in five of six patients after 8 weeks of protocol treatment [128,130].

## 7. Pitfalls and Challenges for Clinical Application of miRNAs

Two phase I clinical trials have provided early data elucidating the efficacy of novel miRNA-based treatment approaches [128,129]. Considering the fact that phase I studies are predominantly comprised of previously heavily treated patients who have partially or fully failed other treatment modalities, the potential of partial treatment response can be high (Table 2). However, in a MRX34 trial, treatment-attributed serious adverse events tended to occur late after the completion of daily MRX34 infusions. The serious adverse events included sepsis, hypoxia, cytokine release syndrome, and hepatic failure, which is a pattern suggestive of immune-mediated toxicity. These adverse events were not observed in the pre-clinical tests with MRX34 in animal models, including non-human primates [129]. These findings suggest that the development of appropriate drug delivery systems for these miRNA-based therapies is essential. The potentially wide-ranging impacts of miRNAs on the regulation of gene expression may result in unexpected side effects in normal cells through nontargeted delivery or unintentional targeting due to the expression of cancer-related antigens. Thus, to avoid “off target” effects, such as systemic immune activation, the effective and specific targeting of miRNA therapeutics to cancer tissues which spares normal tissues is an essential, but as yet unresolved, challenge to overcome.

## 8. Conclusions

To date, miRNAs have been widely known for their key roles in both tumorigenesis and tumor suppression, and have been extensively studied in the field of NSCLC. miRNAs represent a powerful tool to help in the diagnosis, prognosis, and prediction of response to various treatments of NSCLC to improve patient survival rates. miRNA mimics of tumor suppressive miRNAs or miRNA inhibitors (such as Antagomirs) against oncogenic miRNAs may have therapeutic potential. However, despite the potential clinical benefits of miRNA mimics, recent phase I trials have shown unexpected adverse events. Thus, the future development of innovative methods of miRNA delivery will be required to avoid evoking an undesirable immune response.

## Figures and Tables

**Table 2:** miRNA-based Therapy Phase I Clinical Trials in NSCLC.

miRNA	Target	Population	N	DLT	Safety (≥G3, %)	Efficacy	Status	References
MRX34 (NCT01829971)	miR-34	HCC, Melanoma, RCC, NSCLC, SCLC, GIST	85	Hypoxia, thrombocytopenia, neutropenia, thrombocytopenia	SAEs (35), deaths (9), fever (4), chills (14), fatigue (9), back/neck pain (5), dyspnea (5), lymphopenia (18), thrombocytopenia (6), neutropenia (8)	ORR: 4%, SD for ≥4 cycles: 24%	Early closed	[129]
TargomiRs, MesomiR-1 trial (NCT02369198)	miR-16	MPM, NSCLC	27	Infusion-related inflammatory symptoms, coronary ischemia, anaphylaxis, cardiomyopathy, non-cardiac pain	lymphopenia (42), temporal hypophosphatemia (15), increased AST or ALT (19), cardiomyopathy (4), infusion-related inflammatory symptoms (8)	ORR: 5%, SD: 68%, DOR: 32 weeks	Completed	[128,130]

DLT: dose-limiting toxicities; HCC: hepatocellular carcinoma; RCC: renal cell carcinoma; NSCLC: non-small cell lung cancer; SCLC: small cell lung cancer; GIST: gastrointestinal stromal tumor; SAEs: severe adverse events; ORR: objective response rate; SD: stable disease; MPM: malignant pleural mesothelioma; AST: aspartate aminotransferase; ALT: alanine aminotransferase; DOR: duration of the objective response.

## Abbreviations

miRNAs	microRNAs
NSCLC	non-small cell lung cancer
3′-UTRs	3′-untranslated regions
SCLC	small cell lung cancer
LUAD	lung adenocarcinoma
LUSC	lung squamous cell carcinoma
pri-miRNAs	primary miRNAs
pre-miRNAs	premature miRNAs
RNase	ribonuclease
RISC	RNA-induced silencing complex
PABP	poly(A)-binding protein
ceRNAs	competing endogenous RNAs
EMT	epithelial-to-mesenchymal transition
ECM	extracellular matrix
VEGFs	vascular endothelial growth factors
MMPs	matrix metalloproteinases
MET	mesenchymal-to-epithelial transition
tsmiR	tumor suppressive miRNA
oncomiR	oncogenic miRNA
EGFR	epidermal growth factor receptor
ALK	anaplastic lymphoma kinase
TNM	tumor, node, metastasis
AUROC	area under the receiver operating characteristics
